# Enrichment dynamics of *Listeria monocytogenes* and the associated microbiome from naturally contaminated ice cream linked to a listeriosis outbreak

**DOI:** 10.1186/s12866-016-0894-1

**Published:** 2016-11-16

**Authors:** Andrea Ottesen, Padmini Ramachandran, Elizabeth Reed, James R. White, Nur Hasan, Poorani Subramanian, Gina Ryan, Karen Jarvis, Christopher Grim, Ninalynn Daquiqan, Darcy Hanes, Marc Allard, Rita Colwell, Eric Brown, Yi Chen

**Affiliations:** 1 Office of Regulatory Science, Center for Food Safety and Applied Nutrition, Food and Drug Administration, 5001 Campus Drive, College Park, MD 20740 USA; 2 Resphera Biosciences, 1529 Lancaster Street, Baltimore, MD 21231 USA; 3 CosmosID, 155 Gibbs Street, Rockville, MD 20850 USA; 4 Office of Applied Research and Safety Assessment, Center for Food Safety and Applied Nutrition, Food and Drug Administration, 8301 Muirkirk Road, Laurel, MD 20708 USA

**Keywords:** *Listeria monocytogenes*, Enrichment, Ice cream, Microbiota, Co-enriching bacteria, 16S rRNA, Shotgun metagenomics, Next-generation sequencing, NGS, ISO, FDA, USDA, Buffered *Listeria* enrichment broth (BLEB), Half-Fraser broth (HFB), Fraser broth (FB), University of Vermont modified broth (UVM)

## Abstract

**Background:**

Microbiota that co-enrich during efforts to recover pathogens from foodborne outbreaks interfere with efficient detection and recovery. Here, dynamics of co-enriching microbiota during recovery of *Listeria monocytogenes* from naturally contaminated ice cream samples linked to an outbreak are described for three different initial enrichment formulations used by the Food and Drug Administration (FDA), the International Organization of Standardization (ISO), and the United States Department of Agriculture (USDA). Enrichment cultures were analyzed using DNA extraction and sequencing from samples taken every 4 h throughout 48 h of enrichment. Resphera Insight and CosmosID analysis tools were employed for high-resolution profiling of 16S rRNA amplicons and whole genome shotgun data, respectively.

**Results:**

During enrichment, other bacterial taxa were identified, including *Anoxybacillus*, *Geobacillus*, *Serratia*, *Pseudomonas*, *Erwinia*, and *Streptococcus* spp. Surprisingly, incidence of *L. monocytogenes* was proportionally greater at hour 0 than when tested 4, 8, and 12 h later with all three enrichment schemes. The corresponding increase in *Anoxybacillus* and *Geobacillus* spp*.*indicated these taxa co-enriched in competition with *L. monocytogenes* during early enrichment hours. *L. monocytogenes* became dominant after 24 h in all three enrichments. DNA sequences obtained from shotgun metagenomic data of *Listeria monocytogenes* at 48 h were assembled to produce a consensus draft genome which appeared to have a similar tracking utility to pure culture isolates of *L. monocytogenes*.

**Conclusions:**

All three methods performed equally well for enrichment of *Listeria monocytogenes*. The observation of potential competitive exclusion of *L. mono* by *Anoxybacillus* and *Geobacillus* in early enrichment hours provided novel information that may be used to further optimize enrichment formulations. Application of Resphera Insight for high-resolution analysis of 16S amplicon sequences accurately identified *L. monocytogenes*. Both shotgun and 16S rRNA data supported the presence of three slightly variable genomes of *L. monocytogenes*. Moreover, the draft assembly of a consensus genome of *L. monocytogenes* from shotgun metagenomic data demonstrated the potential utility of this approach to expedite trace-back of outbreak-associated strains, although further validation will be needed to confirm this utility.

**Electronic supplementary material:**

The online version of this article (doi:10.1186/s12866-016-0894-1) contains supplementary material, which is available to authorized users.

## Background

Optimization of enrichment methods to culture target pathogens from complex environmental, food and clinical samples is an ongoing challenge. Traditionally, samples are incubated in nonselective and/or selective enrichment broths and then plated onto selective media. Enrichment methods for specific foodborne pathogens will benefit from an improved understanding of the taxonomic diversity and relative abundance of microbiota that co-culture during enrichment. Here, we use culture independent next generation sequencing (NGS) to characterize the microbiome at four hour intervals using three different enrichment methods used for recovery of *Listeria monocytogenes* from naturally contaminated ice cream.

*L. monocytogenes* was first reported in 1926 by Murray, Webb and Swann as the causative agent of illness in >rabbits and guinea pigs in a laboratory breeding unit [1, 2]. Although it was long suspected that food might be a mode of transmission for human listeriosis, it was not until after 1980 that several outbreaks conclusively linked *L. monocytogenes* to foods including, coleslaw, milk, cheese, meat, pâté, and jellied pork tongues [1, 3–6]. Listeriosis outbreaks in the United States over the past five years have been associated with contaminated cheeses [7], stone fruits [8], ice cream [9], cantaloupes [10] and caramel apples [11]. Results of the study presented here were obtained from bacteriological analysis of samples from the 2010 to 2015 listeriosis outbreak with several case-patients linked to milkshakes made from contaminated ice cream. Analysis of *L. monocytogenes* in ice cream samples manufactured in the implicated production line provided information about the prevalence and level of *L. monocytogenes* [12, 13] and activity of *L. monocytogenes* in milkshakes prepared from the ice cream [14]. These analyses did not identify *Listeria* species other than *L. monocytogenes* in the ice cream samples [12].

A variety of enrichment media and methods have been developed for detection of *L. monocytogenes*. The three commonly employed methods examined in this study are as follows:

1) **BLEB** as described in the U.S. Food and Drug Administration (FDA) *Bacteriological Analytical Manual* (BAM): 4 h incubation at 30 °C in buffered *Listeria* enrichment broth (BLEB) without antibiotics, followed by 44 h incubation at 30 °C in BLEB with antibiotics [15]; 2) **HFB-FB** as described in the International Organization of Standardization 11290–1: 24 h enrichment in Half-Fraser broth (HFB) at 30 °C followed by 24 h in Fraser broth (FB) at 37 °C [16]; and 3) **UVM-FB** as described in the U.S. Department of Agriculture (USDA) Microbiological Laboratory Guidebook (MLG): 24 h incubation at 30 °C in University of Vermont modified broth (UVM) for 24 h, followed by 24 h in FB at 37 °C [17].

Once *L. monocytogenes* is detected and isolated from a food or environmental source, pulsed-field gel electrophoresis (PFGE) has been a widely applied method for subtyping isolates. PFGE has been the gold standard for the FDA and the Centers for Disease Control and Prevention (CDC) for foodborne outbreak investigations for over 15 years. Recently however, whole genome sequencing (WGS) has been employed to improve resolution of PFGE for identifying closely related strains. Currently, both PFGE and WGS require isolation of confirmed cultures, which typically requires at least 5 to 7 days for *L. monocytogenes*.

*L. monocytogenes* from the same lot of ice-cream examined here was previously enumerated using an Most Probable Number (MPN) method [12]. All samples from the lot tested positive for *L. monocytogenes* with a geometric mean of 3.35 MPN ^−1^g. To complement enumeration data, we used a metagenomic approach to examine how *L. monocytogenes* and other members of the microbiota of the naturally contaminated ice cream responded to three commonly used enrichment methods (BLEB (FDA), HFB-FB (USDA), UVM-FB (ISO)). Ribosomal RNA amplicons and metagenomic sequence data from time-points every 4 h during a 48 h period were used to describe the taxonomic composition of co-enriching bacterial taxa and provide data to explore whether a hybrid culture/metagenomic approach can be used to source track target pathogens before they are fully isolated.

## Results

### Dynamics of *L. monocytogenes* and co-occurring bacteria

**Fig. 1:**
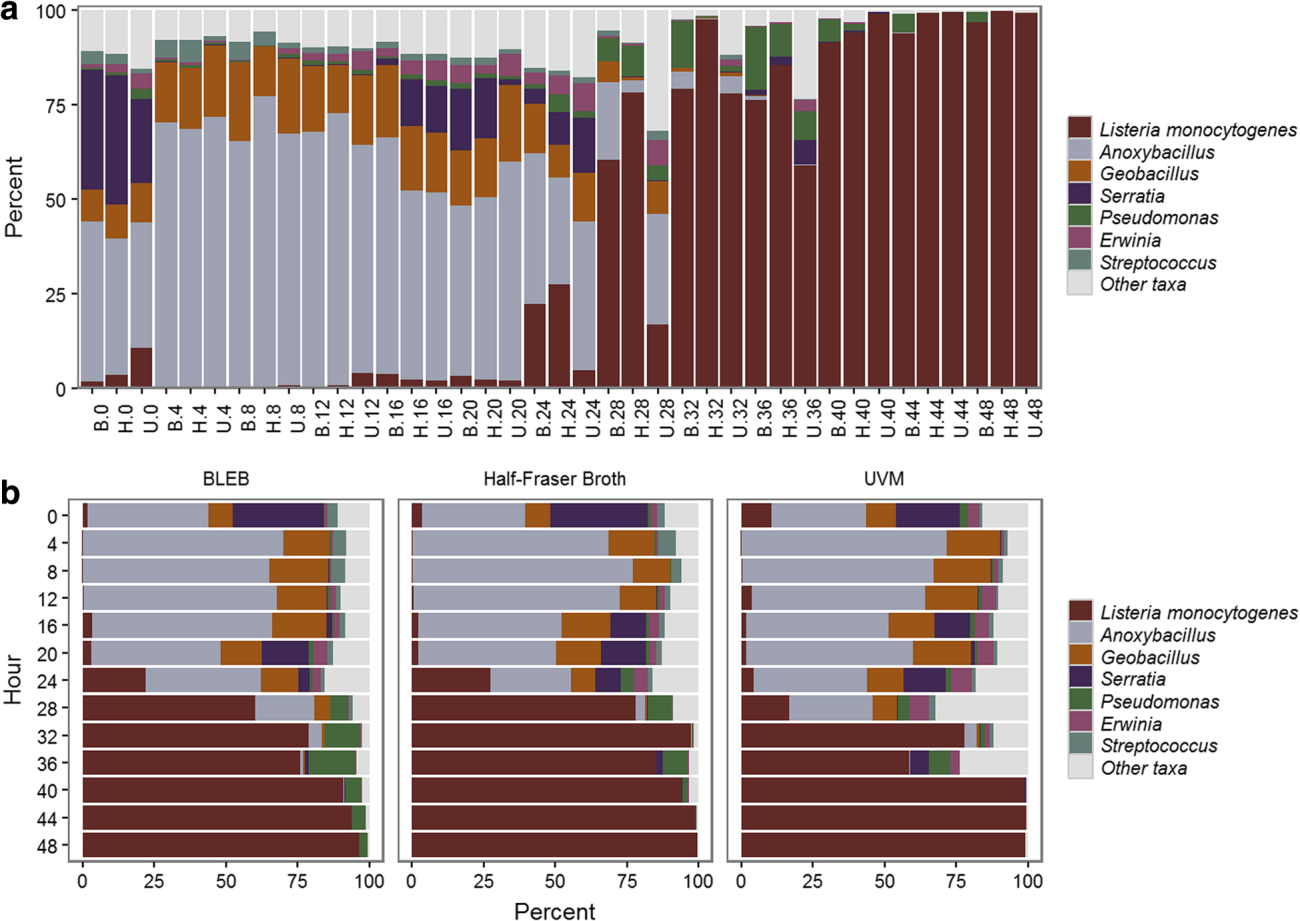
Temporal incidence and relative abundance of *L. monocytogenes* and co-enriching bacterial genera in BLEB, HFB-FB, and UVM-FB media every 4 h for 48 h. Incidence and abundance of *L. monocytogenes* (*maroon*) and co-enriching bacterial genera are shown at four hour intervals from hour 0 through 48 h of enrichment (**a**) for three enrichment protocols (B = BLEB, H = HFB-FB, and U = UVM-FB) (**b**). Four independent replicates for each enrichment at each time-point were pooled and sequenced to constitute each bar. The y-axis shows percent abundance of each taxonomic group within the total library

16S rRNA amplicon sequencing revealed that proportional abundances of *L. monocytogenes* remained low (0–10%) until 24 h of enrichment, even though each enrichment method employed selective antimicrobials (Fig. 1). After 24 h of selective enrichment, relative abundances of *L. monocytogenes* increased at each successive time point until 40 h, at which time relative abundances ranged from 90% to near 100% of the microbial community. Interestingly, *L. monocytogenes* was found to be more abundant at hour 0 than at the three subsequent time points. BLEB and HFB-FB enrichment resulted in higher proportional abundances (although not statistically significant) of *L. monocytogenes* at 24 to 36 h than UVM-FB, while all enrichment yielded equivalent results at later time points.

**Fig. 2:**
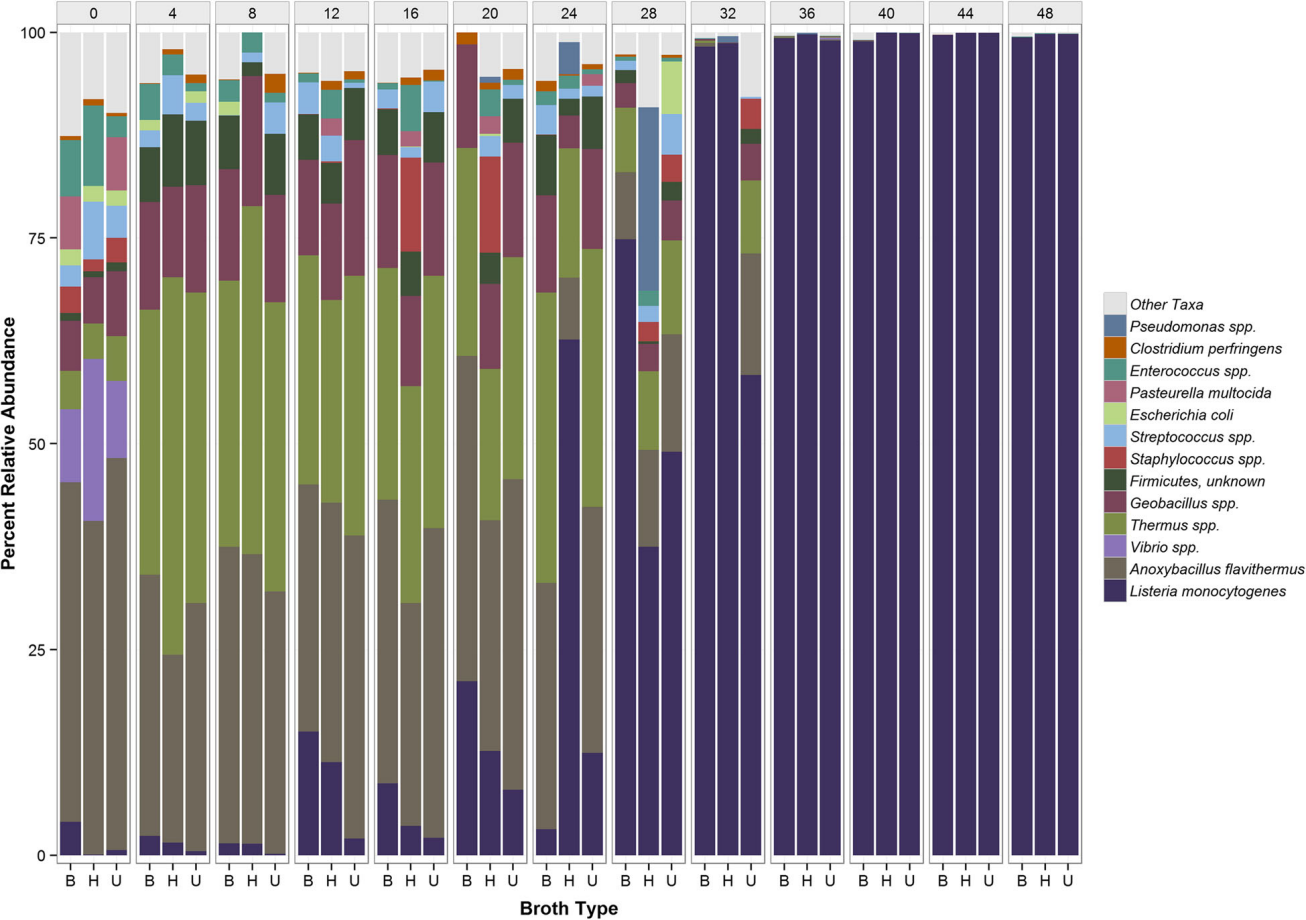
Taxonomic profiles of co-occurring bacteria and *L. monocytogenes* strains derived from the shotgun metagenomic data employing Cosmos ID algorithms. Taxonomy and relative abundance of *L. monocytogenes* and co-occurring bacterial taxa, identified using the k-mer based approached developed by CosmosID

An examination of other community members, using 16S rRNA gene amplicons, revealed a predominance of *Anoxybacillus*, followed by *Serratia*, *Geobacillus*, and *Streptococcus* species (Fig. 1). During enrichment for all three methods (BLEB, UVM-FB, HFB-FB), Bacillaceae genera, *Anoxybacillus* and *Geobacillus* increased rapidly from a combined relative abundance of approximately 45% at hour 0 to almost 90% at hours 4, 8 and 12 (Fig. 1b). Taxonomy based on shotgun sequencing also supported the presence of *Anoxybacillus* and *Geobacillus* species (Fig. 2). Species of both genera are reported as moderately thermophilic and in this study appeared to have an advantage over *L. monocytogenes* during early incubation at 30 °C*.* Additionally, shotgun metagenomic data suggested the presence of two other thermophiles, *Thermus parvatiensis* and *T. thermophilus* (Fig. 2). *Anoxybacillus* spp. have an optimum growth temperature (OGT) ranging from 50 to 62°C and their close relatives, *Geobacillus* spp.*,* have a slightly higher OGT of 55 to 65°C [18]; *Thermus* spp. have an OGT ranging from 50 to 82°C [19, 20].

Another interesting observation of microbial dynamics during enrichment was the change in relative abundance of *Serratia* during the first 24 h of enrichment which appeared to mirror that of *Listeria* (Fig. 1)*.* However, after 24 h of enrichment *Serratia* was outcompeted by other members of the microbial community, mainly *Listeria monocytogenes* in all three enrichment broths. Assuming that hour 0 (initiation of the experiment) resembled the mixed culture microbiota in ice cream, *Serratia* was a well-represented constituent of that microbiota, comprising approximately 20–30%. As observed for *L. monocytogenes*, *Anoxybacillus* and *Geobacillus* spp*.* outcompeted *Serratia* spp. during the 4 to 12 h time points*.* In terms of other taxa present in the ice cream microbiome, relative abundances of *Streptocococcus* and *Erwinia* remained consistent during the first 24 h of enrichment until *L. monocytogenes* began to outcompete the rest of the community, while the relative abundances of *Pseudomonas* species increased after 24 h and decreased after 40 h of enrichment. In the absence of selective antimicrobial agents, we observed a completely different microbial community dynamics during the 48 h of non-selective enrichment (Additional file 2: Figure S2). At 0 to 8 h, we observed a very similar pattern to that in selective enrichments; however, after 16 h of non-selective enrichment, *Bacillus* species dominated the ice cream microbiome, comprising ~80% of the total population. After 24 h, *Lactococcus* emerged and became the slightly dominant species over *Bacillus* for the remainder of the non-selective enrichment.

### *L. monocytogenes* genome coverage by shotgun data

Though 16S rRNA gene sequencing data provided a detailed description of the ice cream microbiome and resulted in the detection of *L. monocytogenes*, a deeper sequence analysis utilizing shotgun metagenomics was necessary to characterize strains of *L. monocytogenes*. Analysis of these samples revealed that near 100% genome coverage can be achieved as early as 24 h of enrichment (Table 1). *L. monocytogenes*-specific sequence reads constituted 0.20 to 0.76% of the metagenome, varying according to enrichment broths employed. At 24 h, we achieved 23 to 97% coverage of the *Listeria* genome at a 7.5× to 12× depth of coverage with 15 to 35 M total metagenomic sequence reads. A similar trend was also observed after 28 h, however, shotgun metagenomic reads from this time point yielded slightly higher genome coverages (44 to 98%) and the depth of coverage ranged from 14.5× to 95×. The highest coverage among these two time points and three enrichment schemes was achieved in BLEB ice cream enrichments at hour 28, which had 45 M total sequence reads, (>95× depth) with near complete genome coverage (>97%), even though the UVM-FB ice cream enrichments contained 82 M total reads (15× depth, 44% genome coverage) (Table 1).

**Table 1:** Shotgun data for potential target assemblies

Broth Type-Hour	*Listeria Genome size (bp)*	Size of the genome covered	*Listeria* read representations	Total sample reads	Predicted depth (x)	% Genome coverage
U-24 h	3109342	738261.44	59165	30102152	12.02	23.74
H-24 h	3109342	2280235.96	115521	15195488	7.6	73.34
B-24 h	3109342	3023679.63	239719	35204116	11.89	97.25
U-28 h	3109342	1360440.77	132749	81801390	14.64	43.75
H-28 h	3109342	3029431.91	419418	20818100	20.77	97.43
B-28 h	3109342	2981548.04	1884038	46165270	94.78	95.89

*B* BLEB, *H* HFB-FB, *U* UVM-FB

Shotgun sequence data are shown for hours 24 and 28 for all three enrichments. The target *L. monocytogenes* genome size is 3,109,342 bases

### Three putative strains of *L. monocytogenes*

**Fig. 3:**
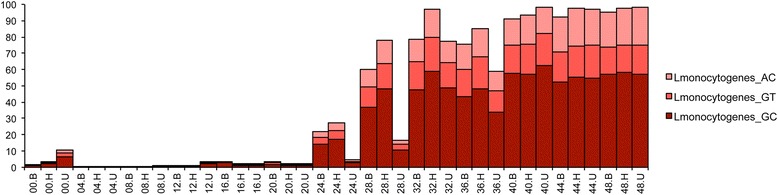
Incidence and abundance of *L. monocytogenes* 16S rRNA gene sequence variants. Relative abundance of three *Listeria monocytogenes* 16S rRNA gene sequence variant types: AC, GT, and GC. Variants occurred at positions 159 and 174 (*E. coli* positions NCBI accession?) within the 16S rRNA gene. The y-axis shows the percent abundance of each *L. monocytogenes*16S rRNA gene sequence variant within the total microbial community at each time point

Interestingly three variants of 16S rRNA gene sequences were observed in *L. monocytogenes* populations from the selective enrichments. Variable nucleotides occurred in the V2 region of the 16S rRNA gene (at positions 159 and 174, *E. coli*) with one variant comprised of AC nucleotides at those positions, a second with GT, and a third, the most abundant, possessing GC (Fig. 3, Table 2). Analysis of 68 closed genomes, available from the PATRIC database, revealed that the majority of *L. monocytogenes* genomes encoded six copies of the 16S rRNA gene (85.3%), with the remaining genomes encoding five (11.8%), or four (5.9%) copies. Six 16S rRNA gene variant sequence motifs were identified, with 96% of the 68 genomes harboring at least one of the variants identified in this study (GC, GT, or AC). Interestingly, among the 68 closed genomes, two sub-variants of type GC were identified (type GC.2 and GC.3), characterized by changes to nucleotides in the G and C allele positions. These minor variants were found in five *L. monocytogenes* lineage I genomes, with type GC.3 comprising the sole 16S rRNA gene sequence variant present in three of these five genomes. Type GC was present in the four lineage III genomes analyzed. Overall, the distribution of major 16S rRNA gene sequence variants was not significantly associated with lineage.

**Table 2:** Characterization and distribution of 16S rRNA variants present in closed *L. monocytogenes* genomes

16S variants across genomes (*n* = 68)					16S variant within genomes						
					Variant by copy number (% total genomes)						
Variant	Genomesa (%)	Lineage (intra-lineal prevalence)				6	5b	4	3	2	1
		I (*n* = 38)	II (*n* = 25)	III (*n* = 4)	Individual variant						
GC	48 (70.6)	26 (68.4)	18 (72)	4 (100)	GC	31 (0.46)	9 (0.13)	5 (0.07)	2 (0.03)	1 (0.01)	n.d.
GT	14 (20.6)	9 (23.7)	5 (20)	n.d.	GT	2 (0.03)	n.d.	n.d.	n.d.	3 (0.04)	9 (0.13)
AC	15 (22.1)	8 (21.1)	6 (24)	n.d.	AC	12 (0.18)	2 (0.03)	n.d.	n.d.	1 (0.01)	n.d.
GC.2	1 (1.5)	5 (13.2)	n.d.	n.d.	GC.2	n.d.	n.d.	n.d.	1 (0.01)	n.d.	n.d.
GC.3	5 (7.4)	1 (2.6)	n.d.	n.d.	GC.3	3 (0.04)	n.d.	1 (0.01)	n.d.	n.d.	1 (0.01)
					Total	48 (0.71)	11 (0.16)	6 (0.09)	3 (0.04)	5 (0.07)	10 (0.15)

^a^Several genomes encoded multiple (different) 16S variants

^b^Six genomes carried only five 16S copies, with types GC and AC found in three and two genomes, respectively

Analysis of intra-genomic distribution of 16S rRNA gene variants (i.e. variant types representing the complete complement of 16S rRNA gene copies within a single genome) revealed the majority of *L. monocytogenes* genomes encoded a single 16S rRNA gene variant type (79.1%), though genomes containing multiple variant types (20.6%) were common. Overall, types GC or AC were most prevalent in genomes encoding a single variant, 51.5 and 20.6%, respectively; while types GC and GT represented the dominant type in mixed 16S rRNA gene variant genomes, 19.1 and 17.6%, respectively. Interestingly, type GT was predominantly found in mixed variant genomes, and accounted for the sole variant in only two genomes, whereas type AC was present in a single mixed variant genome (Table 2).

**Fig. 4:**
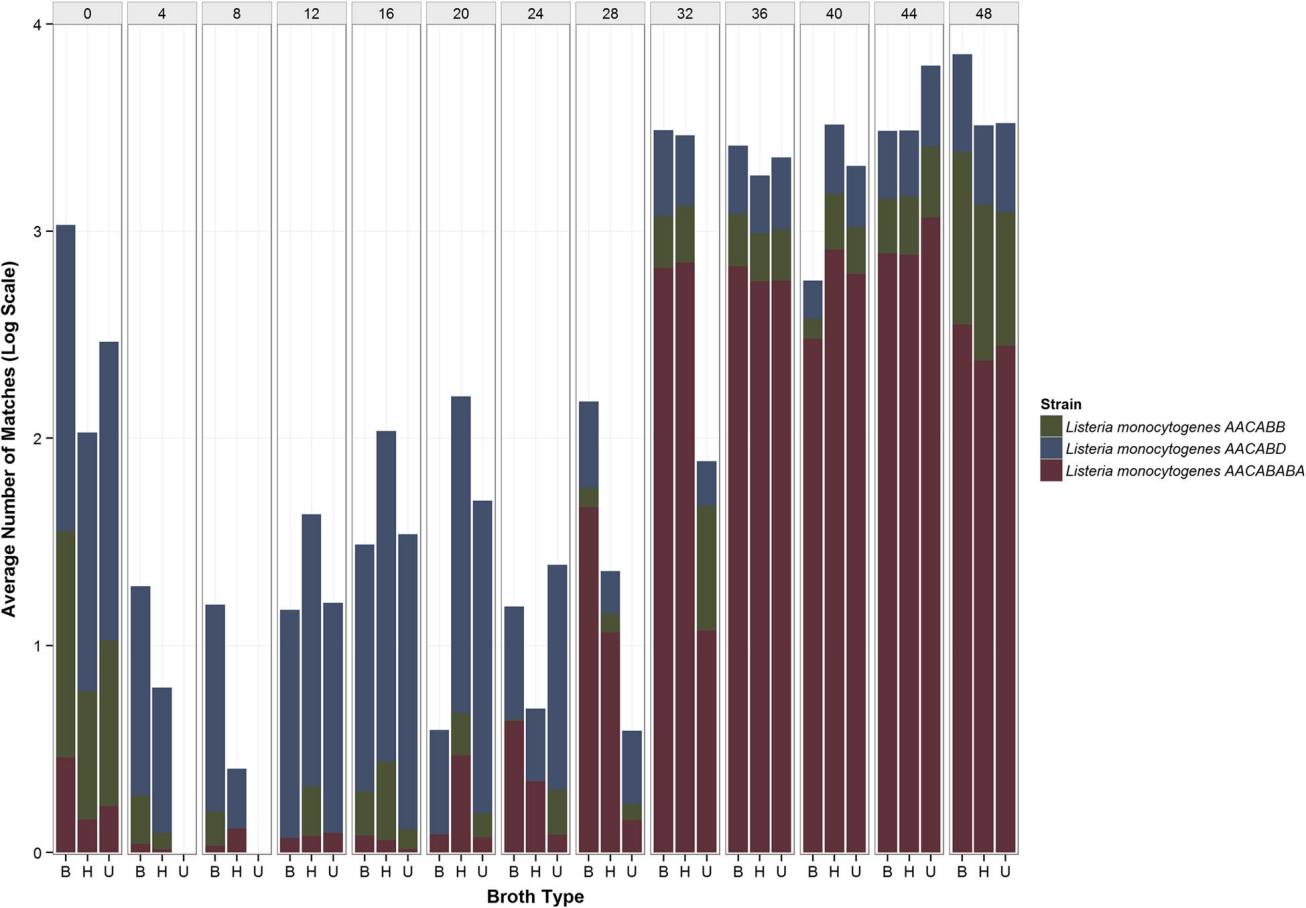
Incidence of *L. monocytogenes* strains detected by Cosmos ID. Relative abundance and incidence of three putative strains of *L. monocytogenes* are shown for hours 0 to 48

It is noteworthy that the AC 16S rRNA gene variant, which was the least abundant of the three types up to hour 40, and less common among the reference set of 68 closed genomes, overtook type GT at hours 44 and 48 across all of the media employed in the study. Type GC remained the most abundant throughout all time points (Fig. 3). This observation was corroborated by analysis of shotgun datasets against a *Listeria* specialty database, suggesting the possibility that of three potentially distinct *L. monocytogenes* strains were present. CosmosID identified strain- specific biomarkers (AACABABA, AACABB, and AACABD) in the metagenomes, which was evidence for three putative *L. monocytogenes* variants (Fig. 4). Furthermore, we observed an interesting strain interplay in terms of their abundance over time where strain AACABD was most abundant during the first 20 h of enrichment and became the least abundant at later time points, due to the rapid upsurge of strain AACABABA, beginning at 28 h (Fig. 4). This same phenomenon was observed in the 16S data which suggests we may be documenting something biologically relevant.

## Discussion

Every food has its own innate or imparted microbiome that will respond to enrichment conditions according to complex eco-physiology. Frequently, antagonistic microorganisms are co-enriched along with target pathogens [21]. For example, *Paenibacillus* spp*.,*which are capable of inhibiting and killing *Salmonella* [22, 23]*,* were also enriched using protocols outlined in the FDA BAM to recover *Salmonella* from tomatoes [21, 24] and cilantro [25]. Thus, continued optimization of reference enrichment protocols is still needed. In the case of *L. monocytogenes,* additional selective agents as well as changes in media formulations may improve efficiency of recovery; however, the situation may be more challenging in this case due to relatedness of *L. monocytogenes* to its co-enriching Bacilli relatives. The increase (almost doubling) of *Anoxybacillus* and *Geobacillus* spp. during the first 8 h of enrichment of the ice cream microbiome suggested these bacteria were able to adapt more readily to the environmental changes inherent to this study (Fig. 1).

Length of lag phase and growth rate typically depend on specific environmental parameters, as well as fitness of the bacterial cells. Many factors have been reported to play a role in length of lag time and/or growth rate for a given bacterial species, including nutritional content, pH, physical environment, inorganic nutrients, temperature, rate of temperature change, water activity, gas atmosphere, inhibitors, spore germination, and initial cell levels, as well as fitness, age, size, and health of individual cells [13, 26, 27]. Although lag time and/or growth rate modeling has been proposed to describe what should occur within a specific set of parameters, such results were variable [26–29]. Hence, it is evident that relationships between growth environments and lag time and growth rate are complex [26], especially for testing foodborne pathogens in food and environmental samples. Additionally, the effect of co-occurring bacterial species in enrichments has not been extensively described or considered in the literature since the analytic tools to do so have not been available and it is not always possible to obtain a sufficient number of naturally contaminated samples that are 100% positive for the target pathogen with relatively homogeneous levels of contamination [12].

The sequence depth and genome coverage achieved in the ice cream metagenomes was sufficient to generate draft *L. monocytogenes* genomes after 24–28 h of enrichment, which is considerably shorter than the 96 h typically required to enrich and isolate viable bacteria from food samples. Near complete genome coverage would facilitate high-resolution phylogenetic analysis, identification of virulence and antibiotic resistance factors, as well as subtyping. Thus this approach might be applicable for rapid and accurate microbial forensics in future work but remains to be more extensively validated.

Type GC represented the predominant allele and was present among the majority of strains belonging to lineage I (808/914 total lineage I strains examined), and was the representative pattern in 30 of the outbreak isolates sequenced in FDA labs. Type GC was also heterogeneously distributed within and between multilocus sequence typing (MLST) clonal groups, designated as clonal complex (CC), for strains within lineage II (361/914 total lineage II strains) Type GC sequences were also present in three lineage III genomes. Type GT and type AC were rare among lineage I (19/914) and lineage II (1/914) strains, respectively. The GC type was significantly prevalent among the human clinical strains from both lineages. The two variable positions (159 and 174) have been shown to contribute to the structure of helix 8, located in the 5′ major domain of the 16S rRNA gene (*E. coli*), which forms direct contact with nearby helices (e.g., helix 6) in the small ribosomal subunit [30]. As correct 16S rRNA gene folding is important for proper ribosome assembly, translational kinetics, and fidelity [31, 32] it is intriguing to consider how specific allelic changes within this region may affect *L. monocytogenes* growth under infection-associated conditions.

## Conclusions

The ability to rapidly and accurately identify the etiologic agent of a foodborne outbreak from a variety of food matrices is critical for preserving public health. Culture-based recovery methods have been used for over 100 years and many have been optimized for recovery of major bacterial and fungal pathogens. We demonstrated that shotgun metagenomic sequencing facilitated the characterization of enrichment microbiota dynamics and identified the presence of competitive species co-occurring during enrichment. As *L. monocytogenes* was enriched, so were other bacterial species. The taxonomy and dynamics of co-enriching species are likely unique to each food commodity and may play a significant, as yet unidentified, role in the recovery and growth of target pathogens. Lag times and growth rates for certain pathogens may be significantly influenced by those co-enriching species “native” to the food commodity or its production environment. Thus, the approach taken in this study, namely to use a culture independent method to describe culture dependent dynamics of *L. monocytogenes* recovery and growth, provides insight and makes it possible to improve both sequence- and culture-based *L. monocytogenes* detection methods. The validation that Resphera Insight can accurately identify *L. monocytogenes* using 16S rRNA amplicons is quite useful. As is the likelihood that draft assemblies of *L. monocytogenes* from shotgun metagenomic data may provide equivalent trace-back utility to WGS genomes - although more work will be needed to confirm this utility.

## Methods

### Ice cream sampling scheme

Ice cream samples (80–85 g /scoop) were aseptically transferred to Whirlpak bags, allowed to stand at room temperature for 20 to 30 min to fully melt. Analytical units were set up as follows: 25 g portions were added to 225 ml of enrichment broth, and stomached for 1 min. Four scoops were used for each of three enrichment protocols.

### Enrichment methods

#### BLEB (FDA BAM)

Ice cream samples were incubated at 30 °C for 48 h in buffered *Listeria* enrichment broth (BLEB) (Cat. CM0897, Thermo Scientific Inc., Waltham, MA). Selective supplements (acriflavin hydrochloride 10 mg/l, nalidixic acid 40 mg/l and cycloheximide 50 mg/l, Cat. SR0149, Oxoid, UK) were added after 4 h of incubation.

#### UVM-FB (USDA)

Ice cream samples were incubated at 30 °C for 24 h in University of Vermont modified broth (UVM) after which, 0.1 ml of each culture was transferred to Fraser broth (FB) and incubated at 37 °C for 24 h. Selective agents were added to UVM and FB prior to enrichment.

#### HFB-FB (ISO)

Ice cream samples were incubated at 30 °C for 24 h in Half-Fraser broth (HFB) after which, 0.1 ml of culture was transferred to FB and incubated at 37 °C for 24 h. Selective agents were added to HFB and FB prior to the enrichment.

For each of the enrichment protocols, 4 ml was taken from each sample at hour 0. After that, every 4 h during the 48 h incubation, enrichments were stomached for 1 min and 4 ml samples taken from each of the four replicates of the three enrichment mixes. Samples were immediately frozen at −20 °C for subsequent DNA analysis.

### DNA extraction

Genomic DNA was extracted using DNeasy Blood and Tissue kit (Cat No. 69506, Qiagen, Germantown, MD, USA) following the protocol for Gram-positive bacteria with minor modification: 1.5 ml of the culture was pelleted (5000 × g, 15 min) and the pellet resuspended in 200 ml of enzymatic lysis buffer containing 20 mM Tris-HCl (pH-8.0), 2 mM Sodium EDTA, 1.2% Triton X- 100, 20 mg/ml of lysozyme. The samples were subsequently incubated for 60 min at 37 °C. DNA was extracted following manufacturer’s protocol.

### Shotgun library preparation

Sequencing libraries were prepared using the Truseq Nano prep kit (Illumina, SanDiego, CA, USA), according to the manufacturer’s specifications.

### 16S rRNA gene amplification

PCR for 16S rRNA gene sequencing targeted the first 330 bases of the V1-V2 region, using the following PCR primers 27 F1 TCGTCGGCAGCGTCAGATGTGTATAAGAGACAGAGAGTTTGATCMTGGCTCAG and 336R1 GTCTCGTGGGCTCGGAGATGTGTATAAGAGACAGACTGCTGCSYCCCGTAGGAGTCT.

PCR cycling conditions consisted of an initial denaturation at 94 °C for 2 min, followed by 25 cycles of 94 °C for 40 s, 56 °C for 15 s, 68 °C for 40 s, and a final extension at 68 °C for 5 min. The amplicons were indexed using Illumina Nextera indexing primers following the manufacturer’s instructions (Illumina, San Diego, CA, USA).

### Sequencing

16S rRNA gene amplicon sequencing was performed on an Illumina MiSeq using the 500 cycle V2 chemistry and cartridges. Shotgun data was obtained by sequencing on an Illumina NextSeq, using V2 chemistry (2 by 150).

### Bioinformatic analyses

#### 16S rRNA amplicon sequence analysis

Raw paired-end reads from the MiSeq platform were merged into consensus fragments by FLASH [33] and subsequently filtered for quality (max error rate 1%) and length (minimum 300 bp) using Trimmomatic [34] and QIIME [35, 36]. Spurious hits to the PhiX control genome were identified using BLASTN and removed. Passing sequences were trimmed of primers, evaluated for chimeras with UCLUST (de novo mode) [37], and screened for chloroplast and mitochondrial contaminants using the RDP classifier [38] with a threshold of 0.5. Sequences were further evaluated for unknown contaminants using a sensitive BLASTN search against the GreenGenes 16S database [39]. High-quality 16S sequences were submitted for high-resolution taxonomic profiling using Resphera Insight (Baltimore, MD, www.respherabio.com). Sequence counts were rarefied to 5000 sequences for each independent replicate for downstream analyses.

### Subtyping of *L. monocytogenes* 16S rRNA gene sequence fragments

To evaluate the potential for multiple cultured strains in the enrichments, all 16S rRNA gene sequences assigned unambiguously to *L. monocytogenes* by Resphera Insight were aligned by PYNAST [36] to a smaller template multiple sequence alignment (MSA) of 5000 randomly selected sequences from the same set (generated by MUSCLE [40]). The full PYNAST MSA was filtered for positions with > 10% gaps and passing positions submitted for entropy calculation [41]. MSA Positions with measured entropy > 0.7 were utilized to assign putative strain membership.

### Validation of Resphera Insight

To perform an external validation of the species level accuracy of Resphera Insight, Resphera Biosciences and Center for Food Safety and Applied Nutrition (CFSAN) scientists collaborated to interrogate 1695 whole-genome shotgun datasets from the GenomeTrakr Project (NCBI Project ID PRJNA183844) designated as *L. monocytogenes* isolates. Raw paired-end sequences were filtered for quality and length, followed by merging of overlapping sequences using FLASH [33]. Merged reads were screened for 16S rRNA fragments using Bowtie2 [42] against a broad database of 16S rRNA gene sequences, with additional BLAST-based filtering to confirm location specific query matches to a reference *L. monocytogenes* 16S rRNA gene (*L. monocytogenes* strain 07PF0776; NCBI accession NR_102780.1).

Passing sequences were submitted to Resphera Insight for high-resolution taxonomic identification. The primary measure of performance was the *Diagnostic True Positive rate* (DTP), defined as the percentage of reads with an unambiguous assignment to *L. monocytogenes* and differences in accuracy associated with changes in read length and gene position were evaluated. We also computed *Sensitivity* (SN), defined as percentage of reads with an unambiguous or ambiguous assignment to *L. monocytogenes.*

Overall, across all 1695 isolates, for fragments ≥200 bp originating within 16S reference gene positions 1–36, Resphera Insight achieved a mean diagnostic true positive rate of 99.43% and a mean sensitivity of 99.94%, with a misassignment rate of 0.06%. We observed increased DTP rates associated with increasing fragment lengths and a loss of species level resolution 3′ to 16S reference gene position 200 (Additional file 1: Figure S1). For fragments <200 bp, we also found a loss of DTP resolution, as more assignments became ambiguous, but sensitivity was not reduced.

To establish benchmark false positive rates of *L. monocytogenes*, we simulated amplicon fragments from our primer region for eight closely related *Listeria* species (*L. fleischmannii, L. grayi, L. innocua, L. ivanovii, L. rocourtiae, L. seeligeri, L. weihenstephanensis, L. welshimeri*). A total of 10,000 sequences per species were simulated, lengths 250–500 bp, with a random nucleotide error rate of 0.5%. Overall, the average sensitivity of detection of these organisms was 99.9%, with a misclassification rate (assignments to *L. monocytogenes*) of 0.07%.

### Shotgun metagenomic data analysis

Unassembled metagenomic sequencing reads were directly analyzed by Genius bioinformatics software package (CosmosID Inc., Rockville, MD), described elsewhere [43, 44] to achieve identification at the species, subspecies, and/or strain level and quantification of relative abundance. Briefly, the system utilizes curated genome databases and a high performance data-mining algorithm to rapidly disambiguate millions of metagenomic sequence reads into discrete microbial taxa. The pipeline has two separable comparators. The first consists of a pre-computation phase and a per-sample computation. The input to the pre-computation phase is a curated reference microbial database and its output is a whole genome phylogeny tree, together with sets of fixed length n-mer fingerprints (biomarkers) that are uniquely identified to distinct nodes of the tree. The second per-sample, computational phase searches the millions of sequence reads against the fingerprint sets. The resulting statistics are analyzed to give fine-grain composition and relative abundance estimates at all nodes of the tree. Overall classification precision is maintained through aggregation statistics.

Furthermore, CosmosID and CFSAN collaboratively developed a specialized *Listeria* database including genome sequences of all *L. monocytogenes* available at the time of this analysis. As for the pre-computational phase describe above, the *L. monocytogenes* database was organized as a phylogenetic tree with thousands of unique and shared biomarkers specific to distinct clades, branches, nodes and leaves of the tree along with specific alphabetical fingerprints reflective of their phylogenetic hierarchies. All metagenomic datasets were analyzed against this *L. monocytogenes* database to investigate the potential presence of multiple strains of *L. monocytogenes* in the ice cream samples. Predicted % genome coverages of *L. monocytogenes* were estimated based on the mean of % total match – a statistics derived from CosmosID algorithm that approximates genome coverage. Predicted depths of coverage were calculated as X = (# of *Listeria* reads X Read length) / (*L. monocytogenes* genome size X Predicted % genome coverage).

## Additional files

Additional file 1: Figure S1. Resphera Insight validation results across 1695 *L. monocytogenes* isolates. (A) Overall, Resphera Insight achieved a mean diagnostic true positive rate of 99.43% and a mean sensitivity of 99.94% with a mis-assignment rate of 0.06%. (B) Evaluation of DTP by read length and gene position. Among reads ≥200 bp covering the first 200 bp of the 16S gene, Resphera Insight achieves up to 99.7% DTP rates. For sequences <200 bp, we find reduced resolution overall. (JPG 38 kb)
Additional file 2: Figure S2. Enrichment protocol without antibiotics did not promote *Listeria* identification. (JPG 38 kb)

## Abbreviations

BLEB: Buffered *Listeria* Enrichment broth
FB: Fraser broth
HFB: Half-Fraser broth
*L. monocytogenes*: *Listeria monocytogenes*
NGS: Next generation sequencing
UVM: University of Vermont modified broth
