# Supplementary figures and images for: Enrichment dynamics of Listeria monocytogenes and the associated microbiome from naturally contaminated ice cream linked to a listeriosis outbreak
Source: BMC Microbiol. 2016 Nov 16;16:275. doi: 10.1186/s12866-016-0894-1 (PMC5112668; doi:10.1186/s12866-016-0894-1)

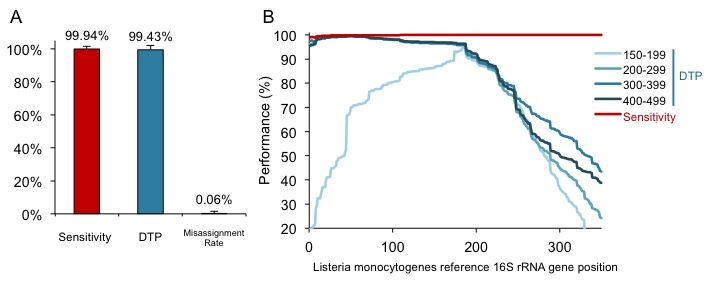

Supplement: Additional file 1: Figure S1. — Resphera Insight validation results across 1695 L. monocytogenes isolates. (A) Overall, Resphera Insight achieved a mean diagnostic true positive rate of 99.43% and a mean sensitivity of 99.94% with a mis-assignment rate of 0.06%. (B) Evaluation of DTP by read length and gene position. Among reads ≥200 bp covering the first 200 bp of the 16S gene, Resphera Insight achieves up to 99.7% DTP rates. For sequences <200 bp, we find reduced resolution overall. (JPG 38 kb) [file 12866_2016_894_MOESM1_ESM.jpg]

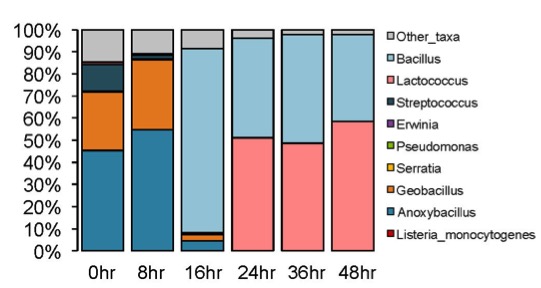

Supplement: Additional file 2: Figure S2. — Enrichment protocol without antibiotics did not promote Listeria identification. (JPG 38 kb) [file 12866_2016_894_MOESM2_ESM.jpg]
